# Adiponectin, Leptin and Resistin in Patients with Psoriasis

**DOI:** 10.3390/jcm12020663

**Published:** 2023-01-13

**Authors:** Sylwia Słuczanowska-Głabowska, Marzena Staniszewska, Mariola Marchlewicz, Ewa Duchnik, Karolina Łuczkowska, Krzysztof Safranow, Bogusław Machaliński, Andrzej Pawlik

**Affiliations:** 1 Department of Physiology, Pomeranian Medical University, 70-111 Szczecin, Poland; 2 Department of Dermatology, Pomeranian Medical University, 70-111 Szczecin, Poland; 3 Department of Aesthetic Dermatology, Pomeranian Medical University, 70-111 Szczecin, Poland; 4 Department of General Pathology, Pomeranian Medical University, 70-111 Szczecin, Poland; 5 Department of Biochemistry and Medical Chemistry, Pomeranian Medical University, 70-111 Szczecin, Poland

**Keywords:** psoriasis, adiponectin, leptin, resistin

## Abstract

Psoriasis is a common chronic, inflammatory skin disease characterised by keratinocyte hyperproliferation, parakeratosis, and T-cell infiltration. Adipose tissue has an endocrine function, producing an abundance of cytokines and adipokines. It has also been described that the major adipokines, leptin, resistin, and adiponectin, may be involved in the pathogenesis of psoriasis. The aim of the study was to examine the plasma levels of adiponectin, leptin, and resistin in patients with psoriasis and their correlations with disease activity parameters: Psoriasis Activity Severity Index (PASI), Dermatology Life Quality Index (DLQI), and Body Surface Area (BSA) index, as well as selected clinical parameters. The study included 53 patients with the plaque type and 31 healthy controls. The plasma concentrations of adiponectin were significantly lower in patients with psoriasis (*p* < 0.001) than in the control group. The plasma concentrations of leptin were higher in patients with psoriasis, however, due to high intra-patient variability of leptin plasma concentrations these differences did not reach statistical significance (*p* = 0.2). The plasma concentrations of resistin were significantly increased in patients with psoriasis compared to healthy controls (*p* = 0.02). There were no statistically significant correlations between adiponectin and leptin plasma concentrations and values of PASI, DLQI, and BSA. The resistin plasma concentrations correlated significantly with DLQI values. Additionally, we examined the correlations between adiponectin, leptin, and resistin plasma concentrations, and selected clinical parameters. Plasma concentrations of adiponectin correlated significantly with CRP values and ALT values. Leptin plasma concentrations correlated significantly with creatinine values. The results of our study confirm the role of adiponectin, leptin, and resistin in the pathogenesis of psoriasis.

## 1. Introduction

Psoriasis is the most common chronic congenital and inflammatory skin disease characterised by keratinocyte hyperproliferation, parakeratosis, and T-cell infiltration with periods of exacerbation and remission [1,2]. Psoriasis has a negative impact on a patient’s physical and emotional quality of life. There are many types of psoriasis, but the most common variety, which affects 85–90% of patients, is plaque psoriasis. Psoriasis affects about 2–3% of the world’s population and occurs equally in men and women. Its incidence varies among ethnic groups, with higher rates observed in Western countries. It is a disease with a genetic basis and polygenic inheritance. Environmental, hormonal, and immunological factors play an important role in the development of psoriasis, but the influence of genetic and polygenic background is emphasised [3,4]. Immunological and even autoimmune phenomena play an important role in the aetiopathogenesis of psoriasis. Psoriasis develops because of chronic interactions between hyperproliferating keratinocytes and infiltrating immune cells. It is known that there is an interaction between autoantigens and autoreactive T lymphocytes [5,6]. Activated keratinocytes and T lymphocytes, and a reduction in the number of antigen-presenting Langerhans cells that migrate in the skin as part of the inflammatory infiltrate, are pathogenically important in the development of psoriasis. Keratinocytes produce many cytokines and are responsible for the expression of cell membrane proteins such as HLA-DR and ICAM-1. Keratinocytes expressing MCH II antigens present superantigens to T cells, especially T helper cells (Th1, Th17, and Th22) [7]. Activated T cells produce large amounts of pro-inflammatory cytokines, derived not only from T cells but from keratinocytes and other immune cells, as well. These cause the skin changes characteristic of psoriasis. In subsequent stages of the disease, clonal proliferation of T cells is observed [8].

Adipose tissue has an endocrine function by producing adipokines and numerous cytokines and chemokines. The systemic consequences of excess adipose tissue also affect the skin, causing changes in its physiology and the development of many dermatological diseases [9,10]. Excess adipose tissue is often associated with venous stasis, lymphedema and an increased incidence of infection. Immune processes are also dysregulated, resulting in an excessive synthesis of pro-inflammatory mediators. They are involved in the development of chronic inflammation in the skin, as well as leading to systemic changes including: dyslipidemia, insulin resistance, diabetes, and cardiovascular disease. Previous studies have confirmed the involvement of adipokines in the development of skin diseases, including psoriasis. A special role is attributed to visceral adipose tissue macrophages. They are responsible for the development of inflammation in adipose tissue and skin and for the synthesis of adipokines and numerous pro-inflammatory mediators that induce the formation of psoriatic lesions. Adipokines are mediators with multidirectional effects. Adipokines can cause metabolic disturbances as well as have a protective effect. They can exacerbate as well as slow the development of psoriatic lesions [11,12]. Adiponectin has been shown to have a protective effect on the development of psoriasis, while leptin and resistin exert pro-inflammatory effects. Adipokines can affect the activity and course of the disease process in patients with psoriasis, as well as various clinical parameters.

The aim of the study was to examine the plasma levels of adiponectin, leptin, and resistin in patients with psoriasis and their correlations with disease activity parameters: Psoriasis Activity Severity Index (PASI), Dermatology Life Quality Index (DLQI) and Body Surface Area (BSA) index, as well as selected clinical parameters.

## 2. Materials and Methods

### 2.1. Patients

Eighty-four subjects were included in the study, 53 with plaque-type psoriasis and 31 healthy controls. Patients were recruited from the Department of Dermatology at Pomeranian University in Szczecin, Poland. Each participant was carefully examined by a dermatologist, who classified psoriasis according to the International Classification of Diseases, Tenth Revision [13]. The diagnosis was established based on clinical features, and disease activity was measured using the Psoriasis Activity Severity Index (PASI), the Dermatology Life Quality Index (DLQI), and the Body Surface Area (BSA) index. These indices were assessed by the same investigator.

Patients included in the study were treated only with topical therapy. Exclusion criteria were systemic treatment of psoriasis (biologics, immunosuppressants, and other oral antipsoriatic drugs) and diabetes, thyroid, adrenal, kidney, liver, gastrointestinal, oncologic, or other autoimmune disease. Information on lifestyle factors and medical history was obtained during clinical evaluation. A detailed history included disease duration, age at diagnosis, family history of psoriasis, and lifestyle factors such as smoking and alcohol consumption. The control group consisted of healthy subjects without diabetes, cardiovascular, thyroid, adrenal, kidney, liver, gastrointestinal, oncological, or autoimmune diseases, as well as obesity. The detailed characteristics of patients and the control group are presented in Table 1 [14].

Venous blood samples (5 mL) were collected using vacutainer tubes. This study was conducted according to the guidelines of the Declaration of Helsinki, and all procedures involving human subjects/patients were approved by the Ethical Committee of Pomeranian University in Szczecin (Poland), number KB-0012/105/17.

### 2.2. Assessment of Adiponectin, Leptin, and Resistin Concentrations in the Plasma of Patients with Psoriasis and Control Subjects

The plasma concentrations of adiponectin, leptin, and resistin were tested in each sample using a magnetic bead-based multiplex assay according to the manufacturer’s procedure (Luminex Assay R&D).

### 2.3. Statistical Analysis

Since the distributions of quantitative variables differed significantly from the normal distribution (Shapiro-Wilk test), non-parametric tests were used. Values were compared between groups with a Mann-Whitney test, and correlations within groups were assessed with the Spearman rank correlation coefficient. A *p*-value of < 0.05 was considered a statistically significant result.

## 3. Results

### 3.1. Assessment of Adiponectin, Leptin and Resistin Plasma Concentrations in Patients with Psoriasis and Control Subjects

**Figure 1:**
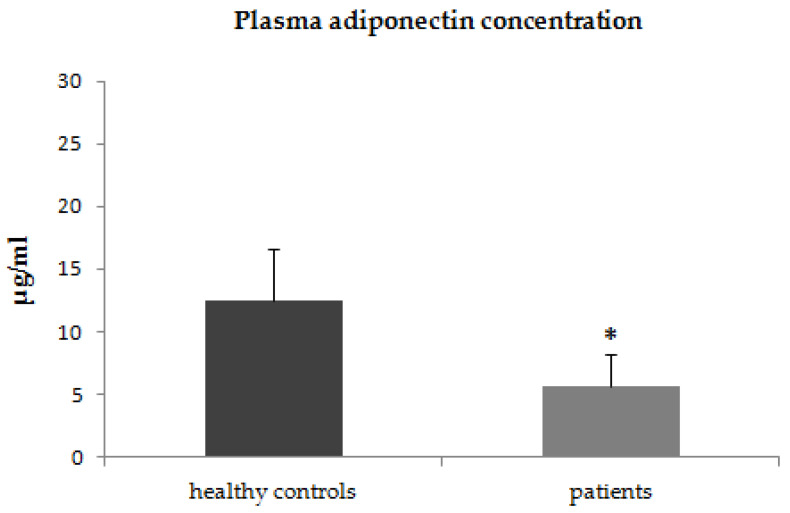
Plasma concentrations of adiponectin in patients with psoriasis and control group * *p* < 0.001.

**Figure 2:**
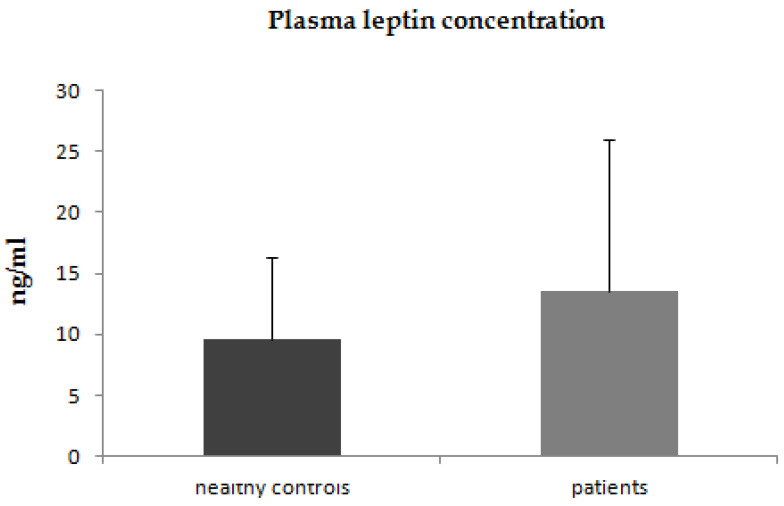
Plasma concentrations of leptin in patients with psoriasis and control group.

**Figure 3:**
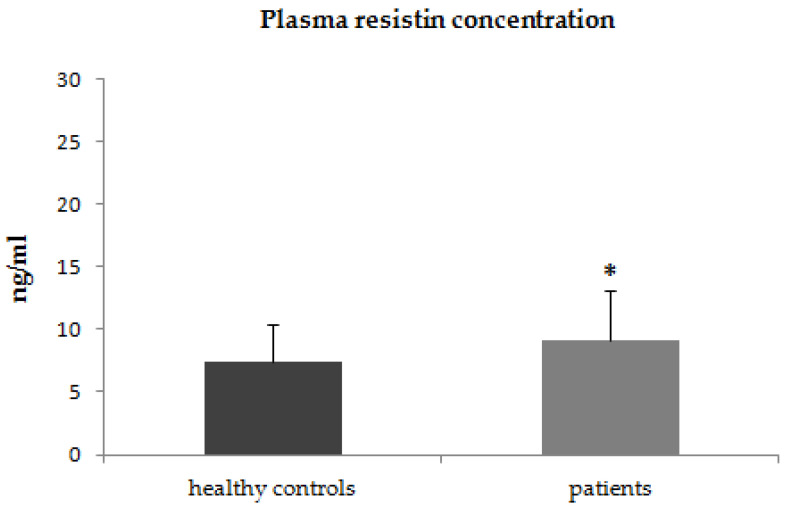
Plasma concentrations of resistin in patients with psoriasis and control group * *p* = 0.02.

In the first step of our study, we compared the plasma concentrations of adiponectin, leptin, and resistin between patients with psoriasis and healthy control subjects. The plasma concentrations of adiponectin were significantly lower in patients with psoriasis (*p* < 0.001) than in the control group (Figure 1). The plasma concentrations of leptin were higher in patients with psoriasis, however, due to high intra-patient variability of leptin plasma concentrations these differences did not reach statistical significance (*p* = 0.2) (Figure 2). The plasma concentrations of resistin were significantly increased in patients with psoriasis compared to healthy controls (*p* = 0.02) (Figure 3).

**Figure 4:**
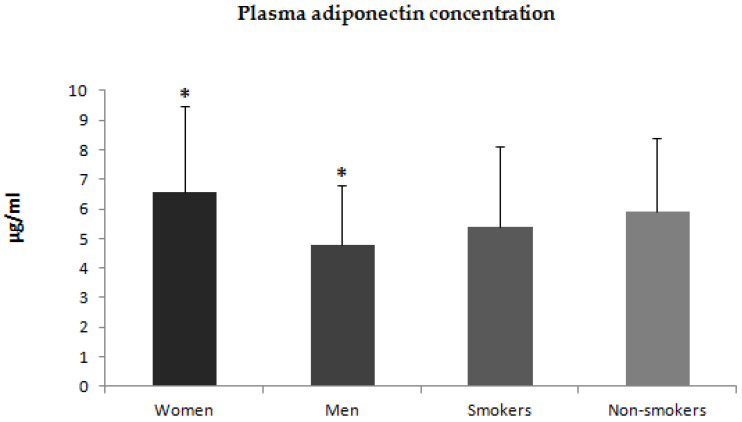
Plasma concentrations of adiponectin in women and men with psoriasis and smoking and non-smoking patients * *p* < 0.001.

**Figure 5:**
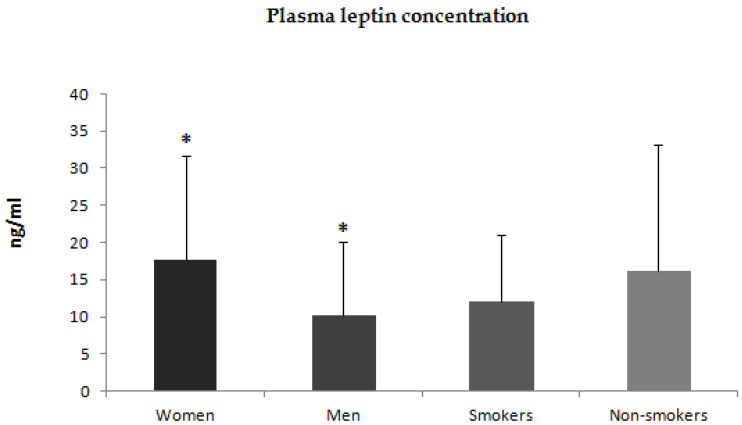
Plasma concentrations of leptin in women and men with psoriasis and smoking and non-smoking patients * *p* = 0.002.

**Figure 6:**
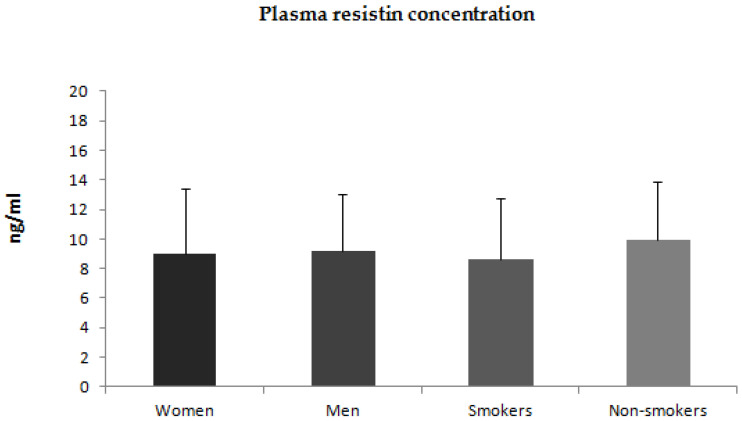
Plasma concentrations of resistin in women and men with psoriasis and smoking and non-smoking patients.

Additionally, we compared plasma concentrations of adiponectin, leptin, and resistin between men and women. Plasma concentrations of adiponectin and leptin were higher in women than in men (Figure 4 and Figure 5). There was no statistically significant difference in plasma concentration of resistin between men and women (Figure 6). We also compared plasma concentrations of adiponectin, leptin, and resistin between smoking and non-smoking patients. These differences were statistically nonsignificant (Figure 4, Figure 5 and Figure 6).

### 3.2. Correlations between Plasma Concentrations of Adiponectin, Leptin and Resistin and Disease Activity Parameters in Patients with Psoriasis

**Figure 7:**
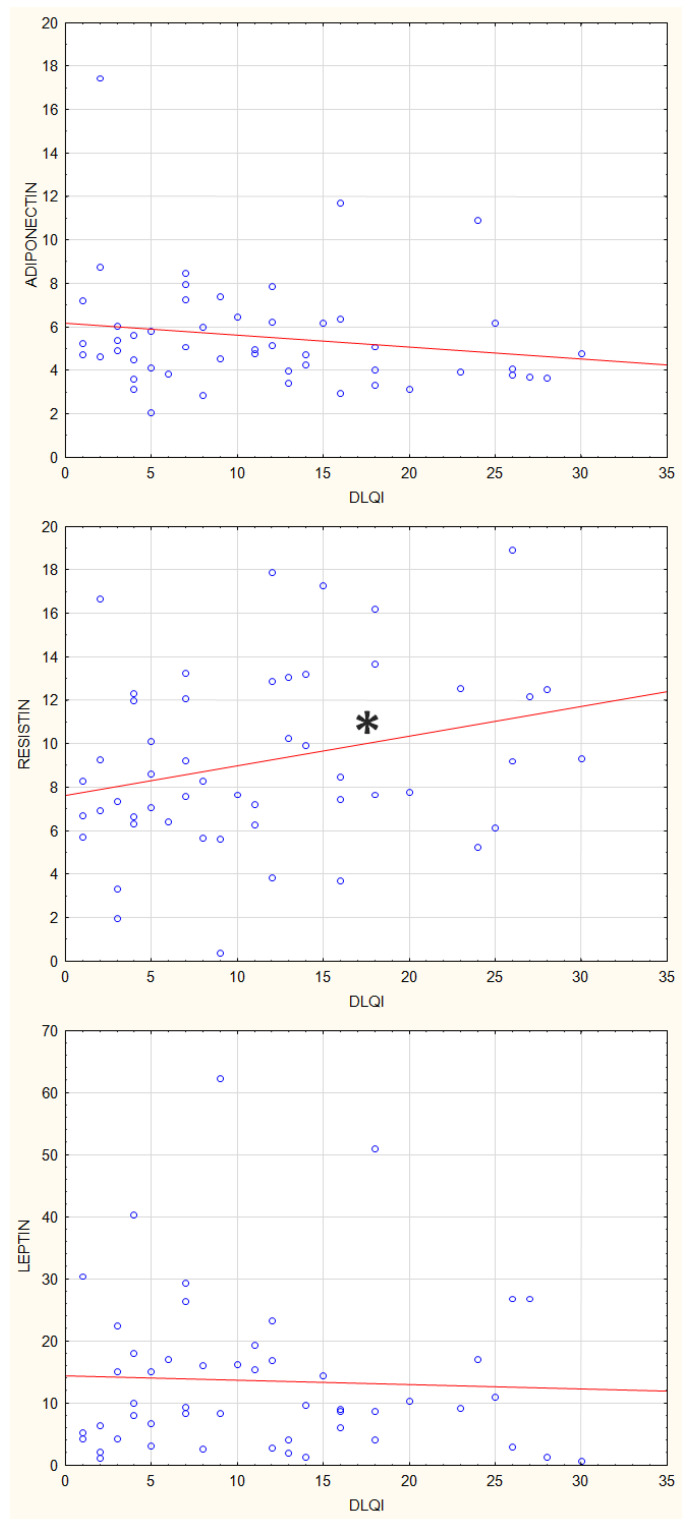
Correlations between plasma concentrations of adiponectin, leptin, and resistin and DLQI index, * *p* = 0.03.

**Figure 8:**
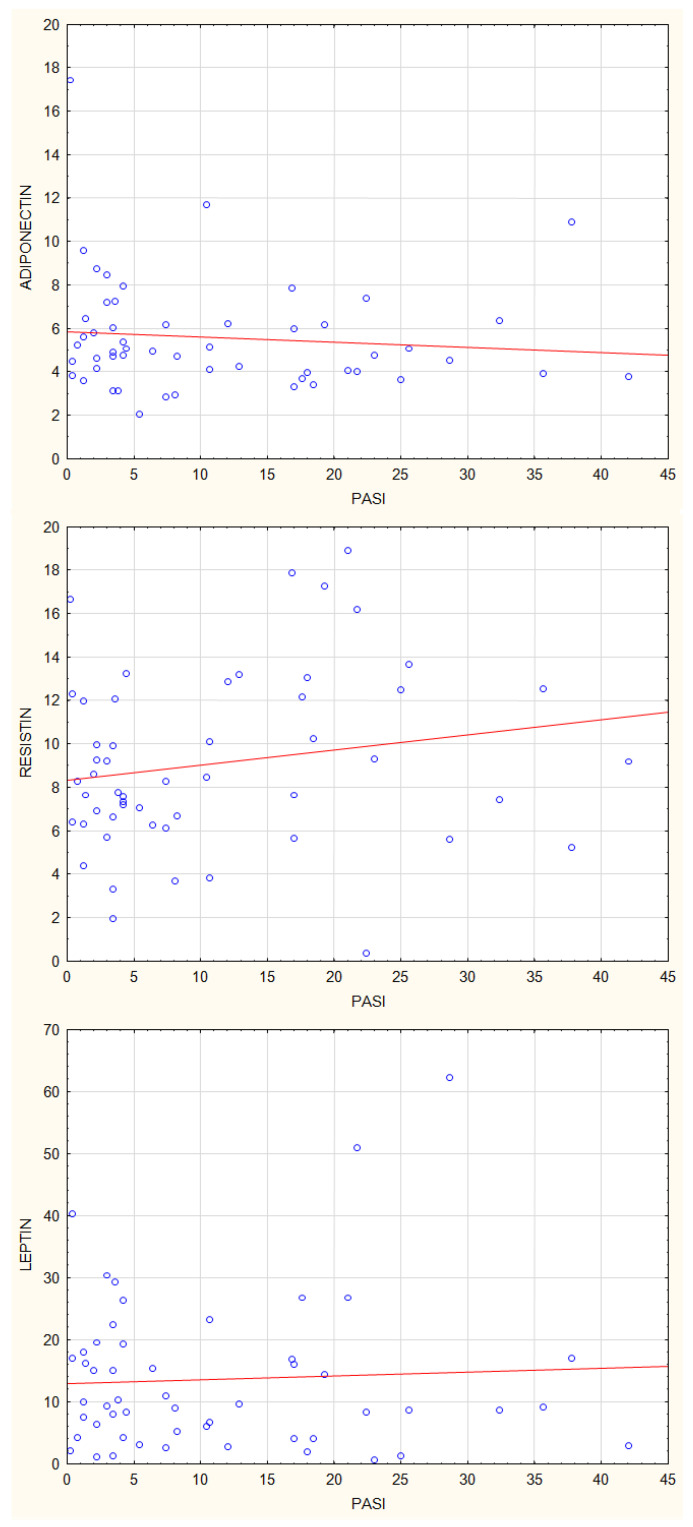
Correlations between plasma concentrations of adiponectin, leptin and resistin and PASI index.

**Figure 9:**
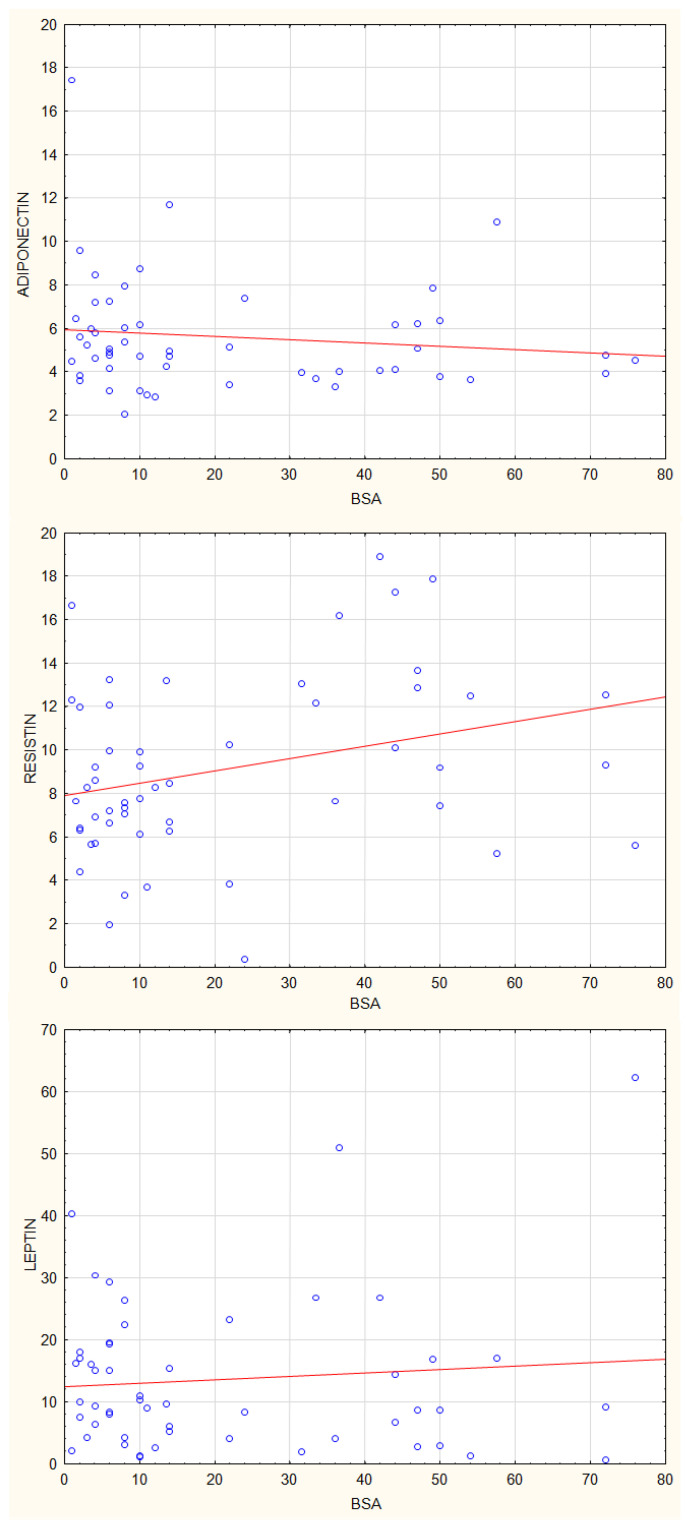
Correlations between plasma concentrations of adiponectin, leptin and resistin and BSA index.

Then we analysed the correlations between plasma concentrations of adiponectin, leptin, and resistin, and parameters of psoriasis activity using the Dermatology Life Quality Index (DLQI), Psoriasis Activity Severity Index (PASI), and Body Surface Area (BSA) index. There were no statistically significant correlations between adiponectin and leptin plasma concentrations and values of, DLQI, PASI and BSA (Figure 7, Figure 8 and Figure 9). The resistin plasma concentrations correlated significantly with DLQI values (*p* = 0.03) (Figure 7).

### 3.3. Correlations between Plasma Concentrations of Adiponectin, Leptin, and Resistin and Clinical Parameters in Patients with Psoriasis

Additionally, we examined the correlations between adiponectin, leptin, and resistin plasma concentrations and selected clinical parameters. As shown in Table 1, plasma concentrations of adiponectin correlated significantly with the age of patients, whereas there were no statistically significant correlations between plasma levels of adiponectin, leptin and resistin and the age of disease diagnosis.

Plasma concentrations of adiponectin correlated significantly with CRP values and ALT values. Leptin plasma concentrations correlated significantly with BMI and creatinine values. There were no statistically significant correlations between plasma concentrations of adiponectin, leptin, and resistin and lipid parameters as well as sodium and potassium plasma concentrations. A positive correlation was found between leptin levels and plasma glucose levels, which had borderline statistical significance (*p* = 0.05) (Table 2).

## 4. Discussion 

Adipose tissue has the very important endocrine functions of secreting adipokines and numerous cytokines [15]. Adipokines have multidirectional effects on the development of such diseases as dyslipidaemia, insulin resistance, diabetes, cardiovascular disease, rheumatoid arthritis, and kidney disease [16]. It has been shown that the action of adipokines can be multidirectional, they can both exacerbate the development of some diseases, as well as inhibit others. Previous studies suggest that adiponectin may have anti-inflammatory effects in psoriasis, slowing the development of the disease, while leptin and resistin may increase inflammation, exacerbating the symptoms of psoriasis [11,12]. Our study examined plasma concentrations of adiponectin, leptin, and resistin in patients with psoriasis. The concentrations of leptin and resistin were higher in patients with psoriasis than in the control group, while adiponectin concentrations were lower. There were no statistically significant correlations between plasma levels of adiponectin and leptin and disease severity parameters (PASI, DLQI, and BSA), while resistin plasma concentrations correlated significantly with DLQI values.

Adipokines affect metabolic processes and appear to provide a pathophysiological link between skin inflammation and metabolic changes in patients with psoriasis [15,16]. Adipokines are secreted by adipocytes and other cells such as macrophages [11,12]. In addition, it has been found that macrophages and monocytes outside adipose tissue, as well as endothelial and epithelial cells, can secrete adipokines [11,12]. Adipokines can exert very multidirectional effects. Resistin contributes to insulin resistance and diabetes. Leptin can lead to uncontrolled eating and weight gain promoting insulin resistance. In turn, adiponectin increases insulin sensitivity, decreasing insulin resistance [11,12].

Leptin increases the synthesis of pro-inflammatory mediators that play an important role in the development of psoriasis, such as TNF-α and CXCL8 [17,18]. These mediators can induce the activation of immune cells involved in the development of inflammation in the skin and psoriatic lesions [19]. The results indicate that adiponectin exerts anti-inflammatory effects in psoriasis by reducing the production of IL-6 and TNF-α [16,20]. On the other hand, TNF-α has been shown to inhibit adiponectin production, which may be one of the factors for reduced adiponectin levels in patients with psoriasis [21]. Studies suggest that adipokines in patients with psoriasis may contribute to the metabolic syndrome, regardless of BMI, and to the development of psoriatic skin lesions [22,23,24,25,26].

Previous studies have suggested that adipokines, may both exacerbate and attenuate skin inflammation in patients with psoriasis, as supported by reports of correlations between adipokines and psoriasis severity [25]. Adiponectin plays an anti-inflammatory role in keratinocytes by inhibiting the synthesis of pro-inflammatory cytokines, while increasing the secretion of anti-inflammatory cytokines [26,27,28,29]. Previous studies have shown lower adiponectin levels in patients with psoriasis than in healthy individuals [30,31,32]. Our results also showed lower levels of adiponectin in patients with psoriasis compared to healthy controls. We also found a positive correlation between plasma adiponectin levels and plasma C-reactive protein (CRP) levels. While the first studies suggested a negative correlation between plasma adiponectin levels and CRP levels, subsequent studies have shown a positive correlation [33,34,35,36]. This is explained by the stimulation of adiponectin synthesis by pro-inflammatory factors such as CRP. In our previous study, we demonstrated the stimulation of adiponectin expression in fibroblast-like synoviocytes by pro-inflammatory mediators such as TNF and LPS [37].

Adiponectin has also been shown to exert its anti-inflammatory effects in psoriasis patients by restoring the balance between Th1-Th17/Th2 and inhibiting IL-17A synthesis [38,39]. The role of adiponectin in the development of psoriasis has also been confirmed in experimental animal models. Mice lacking adiponectin develop severe psoriasis-like dermatitis and elevated levels of the Th17-related cytokines IL-17A, IL-17F, and IL-22 [40]. In our study, we found no correlation of plasma adiponectin setpoint with parameters of psoriasis activity, which is consistent with most clinical observations [30,35], although there are reports of a negative correlation between plasma adiponectin concentrations and PASI score [40,41,42]. Recent studies suggest that adiponectin is involved in sodium excretion [43,44]. In our study, we did not find statistically significant correlations between adiponectin and sodium plasma concentrations.

Previous studies have shown higher plasma leptin levels in psoriasis patients compared to healthy subjects [45,46,47]. In addition, a marked increase in leptin has been found in obese individuals, especially in those with the coexistence of psoriasis and obesity [48,49]. Furthermore, Mitsuyama et al. showed that leptin mRNA expression was significantly increased in subcutaneous adipose tissue of obese subjects with psoriasis compared to those with a normal BMI value [49]. Interestingly, leptin levels in non-obese patients with psoriasis did not differ significantly from non-obese controls. Leptin has been shown to increase the synthesis of pro-inflammatory mediators involved in the pathogenesis of psoriasis, such as IL-1, IL-6, TNF-α, and CXCL8 [50,51]. These mediators stimulate the Th1/Th17 axis, leading to increased IL-17/IL-23 levels [46,47,48,49,50,51,52,53]. The role of leptin in the pathogenesis of psoriasis has also been confirmed in numerous animal model studies. In our study, we observed increased plasma leptin levels in patients with psoriasis, but due to large intra-individual differences, these differences did not reach statistical significance. In addition, plasma leptin levels correlated significantly with BMI values.

Resistin is an adipokine synthesized mainly by macrophages and monocytes in adipose tissue. It has been shown that pro-inflammatory factors can increase its production [54]. Resistin, in turn, can stimulate B lymphocytes to secrete other pro-inflammatory factors that stimulate keratinocyte proliferation and T-cell recruitment to the skin. In addition, resistin can inhibit the proliferation of Foxp3+ regulatory T (Treg) cells in psoriasis [55]. The results of studies suggest that resistin plays an important role in the development of psoriasis by affecting inflammatory factors such as TNF-α and reducing the population of Foxp3+ Treg cells [55]. Previous studies have shown elevated plasma resistin levels in patients with psoriasis, which correlated with disease activity parameters [48,56,57]. Our study also found that elevated plasma resistin levels in patients with psoriasis correlated with the DLQI index.

Previous studies suggest an effect of diet on the development and course of psoriasis. A low-calorie diet has been shown to significantly improve patients’ clinical status and quality of life, as reflected in improvements in PASI and DLQI [58,59]. A low-calorie diet reduces the production of pro-inflammatory leukotriene B4. In addition, there is a decrease in the activity of CD4+ lymphocytes, and an increase in the production of IL-4, which has an anti-inflammatory effect in psoriasis. A low-calorie diet also reduces free radicals and causes a decrease in oxidative stress [60].

Saturated fatty acids have been shown to exacerbate skin inflammation, independent of obesity-related parameters such as increased fat mass, adipokine, and glucose levels. In patients with psoriasis, saturated fatty acid levels correlated with the activity of the disease process [61]. Saturated fatty acids intensify the inflammatory response leading to the activation of keratinocytes and macrophages. Palmitic acid has been shown to activate intracellular signaling pathways in epidermal keratinocytes, leading to increased synthesis of CCL20, CXCL8, and IL-1β [62]. The same processes can also be activated by leptin. Studies suggest that dietary intake, especially of saturated fatty acids, has an impact on the activation of inflammatory processes in the skin [61]. In addition, a low-calorie diet has been shown to increase plasma adiponectin concentrations together with a decrease in leptin concentrations [63].

## 5. Conclusions

In our study, we found increased plasma concentrations of leptin and resistin in patients with psoriasis, while adiponectin levels were significantly lower than in healthy subjects. There was no statistically significant correlation between adiponectin and leptin levels and disease severity parameters (PASI, DLQI, and BSA), though resistin plasma concentrations correlated significantly with DLQI values. The results of our study confirm the role of adiponectin, leptin, and resistin in the pathogenesis of psoriasis.

## Figures and Tables

**Table 1:** Clinical characteristics of patients and control group.

Parameters	Patients with Psoriasis n-53	Control Group n-31
Male	29	17
Female	24	14
Age [years]	49.7 ± 17.6	48.5 ± 14.2
Age of disease onset [years]	34.03 ± 20.08	-
Smoking	19	12
DLQI	11.47 ± 8.19	-
PASI	11.28 ± 10.87	-
BSA	21.33 ± 21.18	-

**Table 2:** Correlations between adiponectin, leptin and resistin plasma concentrations and clinical parameters.

	Adiponectin		Leptin		Resistin	
*Parameter*	Rs	*p*	Rs	*p*	Rs	*p*
Age	0.28	0.03	0.02	0.83	0.2	0.14
Age of disease onset	0.05	0.71	0.05	0.67	−0.06	0.62
BMI	−0.33	0.14	0.56	0.01	0.24	0.29
Leukocytes	0.02	0.92	0.21	0.33	0.22	0.29
ESR	0.35	0.09	0.36	0.08	0.12	0.58
CRP	0.43	0.04	0.27	0.20	0.33	0.11
AST	−0.22	0.29	0.12	0.58	0.10	0.62
ALT	−0.44	0.03	0.17	0.41	0.10	0.64
Creatinin	0.03	0.98	0.45	0.03	0.09	0.66
Sodium	0.08	0.74	0.13	0.57	0.08	0.73
Potassium	0.26	0.27	0.19	0.41	0.06	0.79
Cholesterol	−0.23	0.28	0.01	0.95	0.30	0.16
LDL	−0.40	0.07	0.05	0.83	0.01	0.97
Triglycerides	−0.02	0.94	0.29	0.19	−0.01	0.97
Glucose	−0.02	0.92	0.43	0.05	0.24	0.29

## Data Availability

Not applicable.
